# Poor Growth and Pneumonia Seasonality in Infants in the Philippines: Cohort and Time Series Studies

**DOI:** 10.1371/journal.pone.0067528

**Published:** 2013-06-28

**Authors:** Stuart Paynter, Robert S. Ware, Marilla G. Lucero, Veronica Tallo, Hanna Nohynek, Eric A. F. Simões, Philip Weinstein, Peter D. Sly, Gail Williams, the ARIVAC Consortium

**Affiliations:** 1 School of Population Health, University of Queensland, Brisbane, Queensland, Australia; 2 Research Institute for Tropical Medicine, Department of Health, Muntinlupa City, Metro Manila, Philippines; 3 Department of Vaccines and Immune Protection, National Institute for Health and Welfare, Helsinki, Finland; 4 University of Colorado Denver School of Medicine, and Colorado School of Public Health, Aurora, Colorado, United States of America; 5 Barbara Hardy Institute, University of South Australia, Adelaide, South Australia, Australia; 6 Children’s Health and Environment Program, Queensland Children’s Medical Research Institute, University of Queensland, Brisbane, Queensland, Australia; National Institutes of Health, United States of America

## Abstract

Children with poor nutrition are at increased risk of pneumonia. In many tropical settings seasonal pneumonia epidemics occur during the rainy season, which is often a period of poor nutrition. We have investigated whether seasonal hunger may be a driver of seasonal pneumonia epidemics in children in the tropical setting of the Philippines. In individual level cohort analysis, infant size and growth were both associated with increased pneumonia admissions, consistent with findings from previous studies. A low weight for age z-score in early infancy was associated with an increased risk of pneumonia admission over the following 12 months (RR for infants in the lowest quartile of weight for age z-scores 1.28 [95% CI 1.08 to 1.51]). Poor growth in smaller than average infants was also associated with an increased risk of pneumonia (RR for those in the lowest quartile of growth in early infancy 1.31 [95%CI 1.02 to 1.68]). At a population level, we found that seasonal undernutrition preceded the seasonal increase in pneumonia and respiratory syncytial virus admissions by approximately 10 weeks (pairwise correlation at this lag was −0.41 [95%CI −0.53 to −0.27] for pneumonia admissions, and −0.63 [95%CI −0.72 to −0.51] for respiratory syncytial virus admissions). This lag appears biologically plausible. These results suggest that in addition to being an individual level risk factor for pneumonia, poor nutrition may act as a population level driver of seasonal pneumonia epidemics in the tropics. Further investigation of the seasonal level association, in particular the estimation of the expected lag between seasonal undernutrition and increased pneumonia incidence, is recommended.

## Introduction

Pneumonia is a major cause of child mortality globally, with most deaths occurring in the tropics [1]. In contrast to temperate settings, where pneumonia and respiratory syncytial virus (RSV) incidence peaks in winter, in many tropical settings yearly pneumonia and RSV epidemics occur during the rainy season [2], [3], [4]. One possible driver of pneumonia seasonality in the tropics is undernutrition. There is consistent evidence from many settings demonstrating that malnourished children have an increased risk of pneumonia [5], [6], [7], [8], [9], and also RSV [10], and in many tropical settings the rainy season is a period of poor nutrition, referred to as the hungry season [11]. Thus it is plausible that seasonal periods of poor nutrition may be a driver of the seasonal peaks of pneumonia.

We have investigated the association between undernutrition and pneumonia incidence in the tropical setting of the Philippines. In this paper we have three aims: A) to confirm that poor nutrition is a risk factor for pneumonia in individual children in this setting, B) to assess the seasonal pattern of nutritional status in the child population in this setting, and C) to assess whether seasonal patterns of nutritional status could be driving yearly pneumonia and RSV epidemics in children in this setting.

### Ethics Statement

The trial “Effectiveness of an 11-valent pneumococcal conjugate vaccine against pneumonia in Philippine children: A double-blind, placebo-controlled, randomised, multicentre, effectiveness study” was approved and monitored by the Ethical and Institutional Review Board of the Research Institute for Tropical Medicine (RITM), the Philippines. Written informed consent was obtained from parents/guardians prior to enrolling infants into the trial. In a small minority of cases both parents were illiterate. In these instances a witness independent of the research team (a relative or neighbour) attested to (1) that the information provided to the parent was from the information sheet, (2) that the parent was given the opportunity to ask questions, and (3) that the parent provided verbal consent for the child to participate in the study. The witness then signed the consent form on behalf of the parent. The Ethical and Institutional Review Board of the RITM approved the consent protocol and information sheets. This retrospective analysis used de-identified data collected during the trial. Ethical approval for the analysis in this paper was granted without the need to seek additional consent, by the University of Queensland School of Population Health Research Ethics Committee in June 2011 (Ethics approval SP070711).

## Methods

The data used in this study were collected during a randomised controlled trial to investigate the efficacy of pneumococcal conjugate vaccine (PCV) in Bohol Province in the Philippines between July 2000 and December 2004 [12]. Three doses of either PCV or saline placebo were administered to the 12,194 trial participants at median ages of 1.8, 2.9 and 3.9 months. Trial participants were weighed at each of these vaccinations. Hospital admissions for respiratory infection were assessed and recorded in all children living in the trial municipalities (whether or not they were trial participants). In our analyses of pneumonia cases we included all hospital admissions with a hospital discharge code for pneumonia (this classification also contains some cases of bronchiolitis). In addition we have performed a seasonal level analysis of admissions due to confirmed RSV infection [13]. The seasonality of both is similar.

### A. Individual Level Analysis of the Association between Nutritional Status and Pneumonia Incidence in Infants

Previous individual level studies have used one of two methods to assess nutritional status: either a single point measure of nutritional status [5], [7], or a measure of growth between two points in time [8], [14]. In our study we have used both approaches. We examined the incidence of pneumonia admissions (survival until first pneumonia admission) according to a point measures of nutritional status (weight for age z-score at third vaccination) and also according to growth between first and third vaccination (weight for age z-score at third vaccination minus the weight for age z-score at first vaccination). Weight for age z-scores were calculated using the WHO child growth standards [15]. In all of these analyses we followed up children from third vaccination, for 12 months in order to reduce confounding by season of follow up. We used multivariable Cox regression for statistical significance tests, including terms for the number of children in the household and vaccination status (PCV or placebo). In addition, when assessing the effect of growth between first and third vaccination on pneumonia incidence, we stratified the analysis according to weight for age z-score at first vaccination (infants with a high z-score at first vaccination were more likely to have negative growth to third vaccination, while infants with a low z-score at first vaccination were more likely to have positive growth, suggesting a degree of endogenous regression to the mean following birth). For all analyses we focussed on the infants with poorest nutritional status when estimating hazard ratios, comparing the rate of pneumonia in infants in the lowest quartiles of nutritional status with the remaining infants.

### B. Assessing the Seasonal Pattern of Nutrition and Growth in the Study Population

We constructed time series of two growth indices to assess seasonality. For the first time series we used weight for age z-score at first vaccination. Weight for age z-score at first vaccination appeared strongly influenced by birth weight: there was a strong individual level correlation between weight for age z-score at first vaccination and at third vaccination (r = 0.75). We therefore considered weight for age z-score at first vaccination to be a proxy measure of birth weight, and used this data to construct a time series of weekly mean birth weight z-scores (attributing each infant’s weight for age z-score at first vaccination to their birth date rather than the date of first vaccination). We used data from all children in the trial who were weighed at first vaccination (n = 12,191). To assess the validity of this time series, we compared it to a monthly time series of the incidence of low birth weight in Bohol between 1979 and 1990, using data collected as part of a case control study examining risk factors for child mortality (n = 3663) [16]. For the second time series we used growth between first and third vaccination. We plotted each infant’s growth (weight for age z-score at third vaccination minus the weight for age z-score at first vaccination) at the midpoint between first and third vaccination, and calculated the mean of these data points for each week of the time series. For this second time series we used data from infants with a weight for age z-score below the median at first vaccination, as only in these infants was our growth measure associated with pneumonia risk (see Results). 5412 of these infants had weight data for first and third vaccination and contributed to the time series.

We also assessed the seasonality of nutrition more directly using quarterly palay (unhusked rice) production in Bohol, available from the Philippines Bureau of Agricultural Statistics [17]. In Bohol 53% of energy intake and 37% of protein intake is provided by rice, making this a good proxy for protein energy nutrition [18]. Seasonal undernutrition typically peaks just prior to the harvest season, when food availability is lowest, and food prices highest [11].

### C. Analysis of the Population Level Association between Growth and Pneumonia Seasonality

We compared weekly pneumonia (n = 1571) and RSV (n = 245) admissions to the seasonality of birth weight, infant growth, and palay production over the period from July 2000 to September 2003. For birth weight seasonality we used the three month moving average of weekly mean birth weight z-scores. We have used three month moving averages in order to smooth out short term fluctuations, in order to concentrate on seasonal scale fluctuations (for any particular week, the three month moving average is the average of that week, the six weeks prior, and the six weeks after, giving an average over 13 weeks). For infant growth we used the three month moving average of weekly mean growth between first and third vaccination. We removed year on year trend from this infant growth time series by subtracting the 12 month moving average (giving a time series of relative infant growth according to season). For palay production seasonality we used the quarterly data. We compared these seasonal scale time series to weekly pneumonia and RSV admissions using cross-correlation analysis, and performed pairwise correlation at points of maximum cross-correlation to assess statistical significance [19].

## Results

### A. Individual Level Analysis of the Association between Nutritional Status and Pneumonia Incidence in Infants

**Figure 1:**
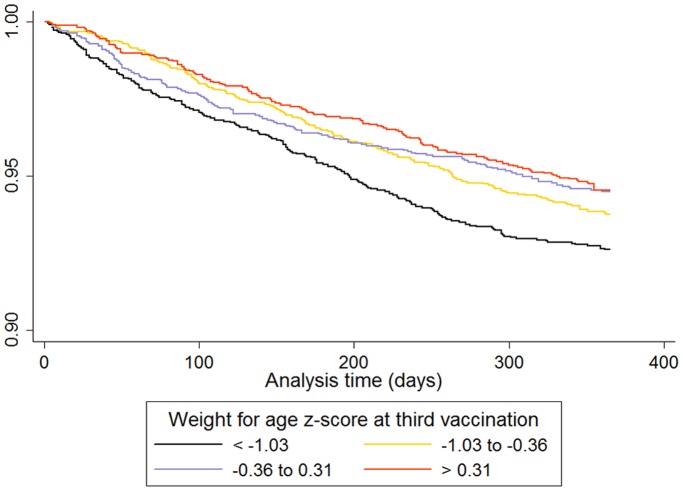
Survival curve (until first pneumonia admission) in children according to quartiles of weight for age z-score at third vaccination.

**Figure 2:**
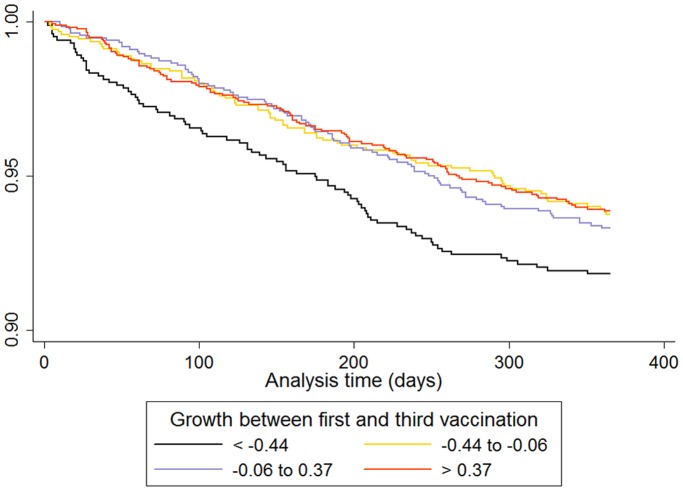
Survival curve (until first pneumonia admission) in children according to quartiles of growth between first and third vaccination, in those children with a weight for age z-score less than the median at first vaccination.

Infants in the lowest quartile of weight for age z-score at third vaccination had a significantly increased rate of pneumonia admission over the following 12 months (Table 1). The survival curve (to first pneumonia admission) for quartiles of weight for age z-score at third vaccination is given in Figure 1. Infants in the lowest quartile of growth between first and third vaccination had a significantly increased rate of pneumonia admission, but only in infants who were smaller than the median at first vaccination (Tables 2 and 3). The survival curve (to first pneumonia admission) for quartiles of growth between first and third vaccination in infants with weight for age z-scores below the median at first vaccination is given in Figure 2.

**Table 1:** Individual level association between weight for age z-score at third vaccination and pneumonia admission.

	Univariate analysis		Multivariate analysis	
	Hazard Ratio	95% CI	Hazard Ratio	95% CI
Weight for age z-score at third vaccination (lowest quartile/other quartiles)	1.31	1.11 to 1.55	1.28	1.08 to 1.51
Number of children in the household (per extra child)	1.12	1.08 to 1.16	1.12	1.09 to 1.16
Vaccination status (PCV/placebo)	0.92	0.80 to 1.07	0.90	0.77 to 1.05

**Table 2:** Individual level association between growth from first to third vaccination and pneumonia admission, in children with a weight for age z-score less than the median at first vaccination.

	Univariate analysis		Multivariate analysis	
	Hazard Ratio	95% CI	Hazard Ratio	95% CI
Growth between first and third vaccination (lowest quartile/other quartiles)	1.32	1.03 to 1.69	1.31	1.02 to 1.68
Number of children in the household (per extra child)	1.12	1.08 to 1.16	1.13	1.08 to 1.18
Vaccination status (PCV/placebo)	0.92	0.80 to 1.07	0.92	0.75 to 1.13

**Table 3:** Individual level association between growth from first to third vaccination and pneumonia admission, in children with a weight for age z-score greater than the median at first vaccination.

	Univariate analysis		Multivariate analysis	
	Hazard Ratio	95% CI	Hazard Ratio	95% CI
Growth between first and third vaccination (lowest quartile/other quartiles)	1.03	0.81 to 1.32	1.04	0.81 to 1.32
Number of children in the household (per extra child)	1.12	1.08 to 1.16	1.12	1.06 to 1.17
Vaccination status (PCV/placebo)	0.92	0.80 to 1.07	0.87	0.70 to 1.09

### B. Assessing the Seasonal Pattern of Nutrition and Growth in the Study Population

**Figure 3:**
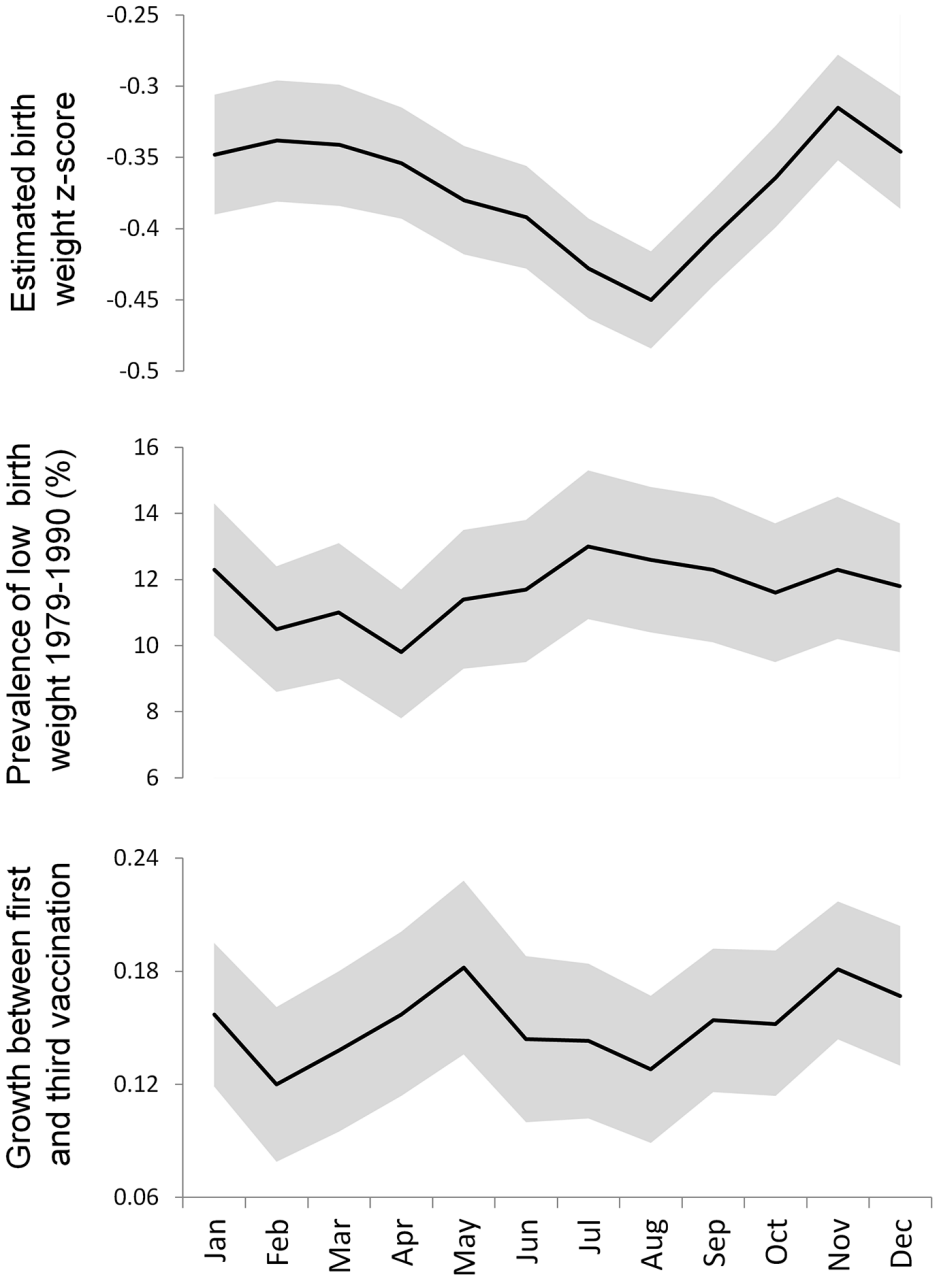
Comparison of seasonal patterns (three month moving averages) of indices of child growth in Bohol. Shaded areas indicate 95% confidence intervals.

Figure 3 shows the comparison of the three month moving averages of the indices of child growth by calendar month (estimated birth weight z-scores, incidence of low birth weight from the 1979–1990 study, and infant growth between first and third vaccination). All three indices indicate poor growth occurs in the third calendar quarter. The time series of infant growth between first and third vaccination also indicates a second period of poor growth in young infants early in the calendar year.

### C. Analysis of the Population Level Association between Growth and Pneumonia Seasonality

**Figure 4:**
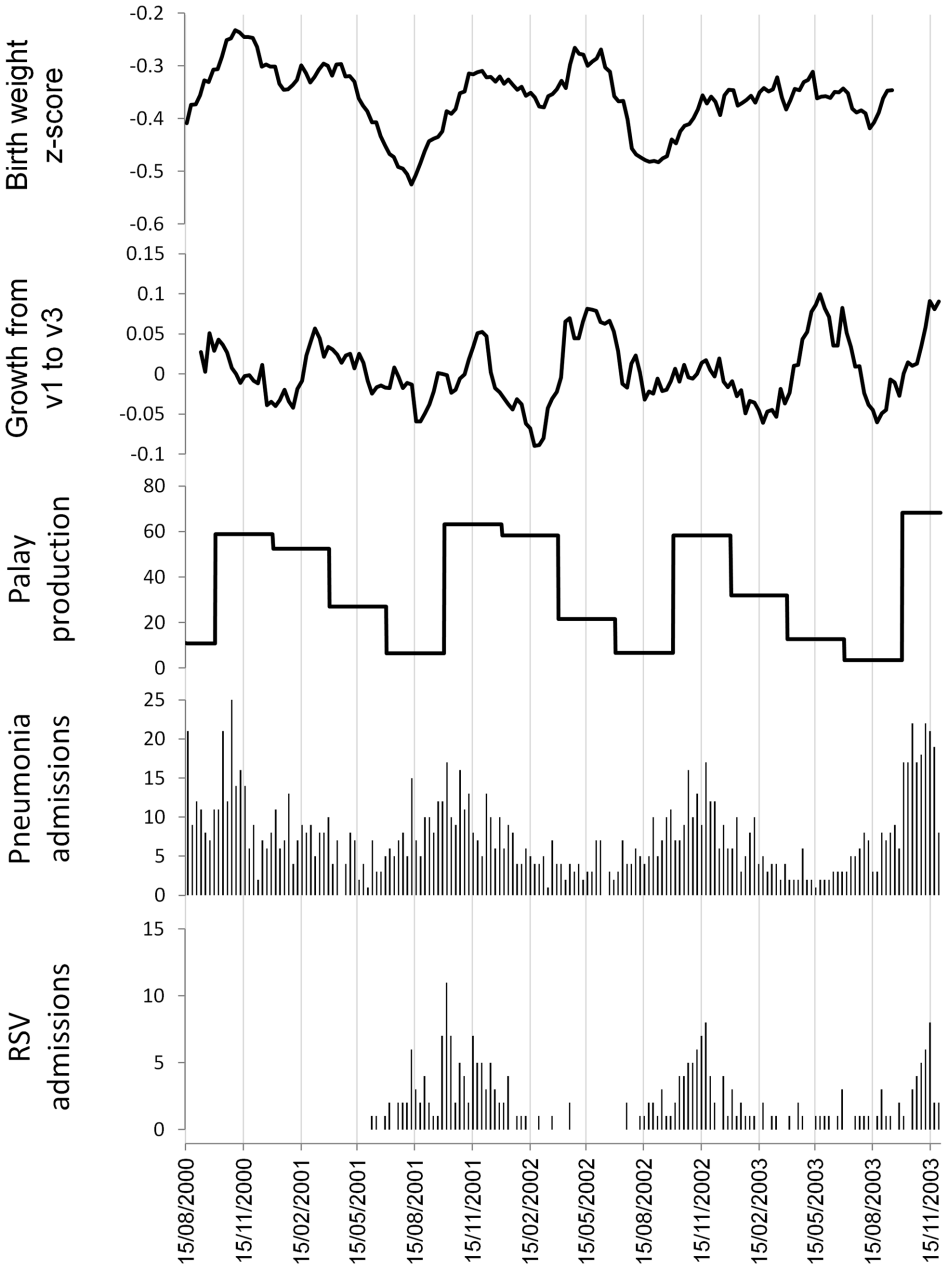
Seasonal patterns (three month moving averages) of estimated birth weight z-scores, relative growth between first and third vaccination, and palay production, together with weekly pneumonia and RSV admissions. Top to bottom: estimated birth weight z-scores, growth between first and third vaccination, palay production (thousands of metric tons), pneumonia admissions in children aged less than 18 months, RSV admissions. Bohol, Philippines 2000 to 2003.

**Figure 5:**
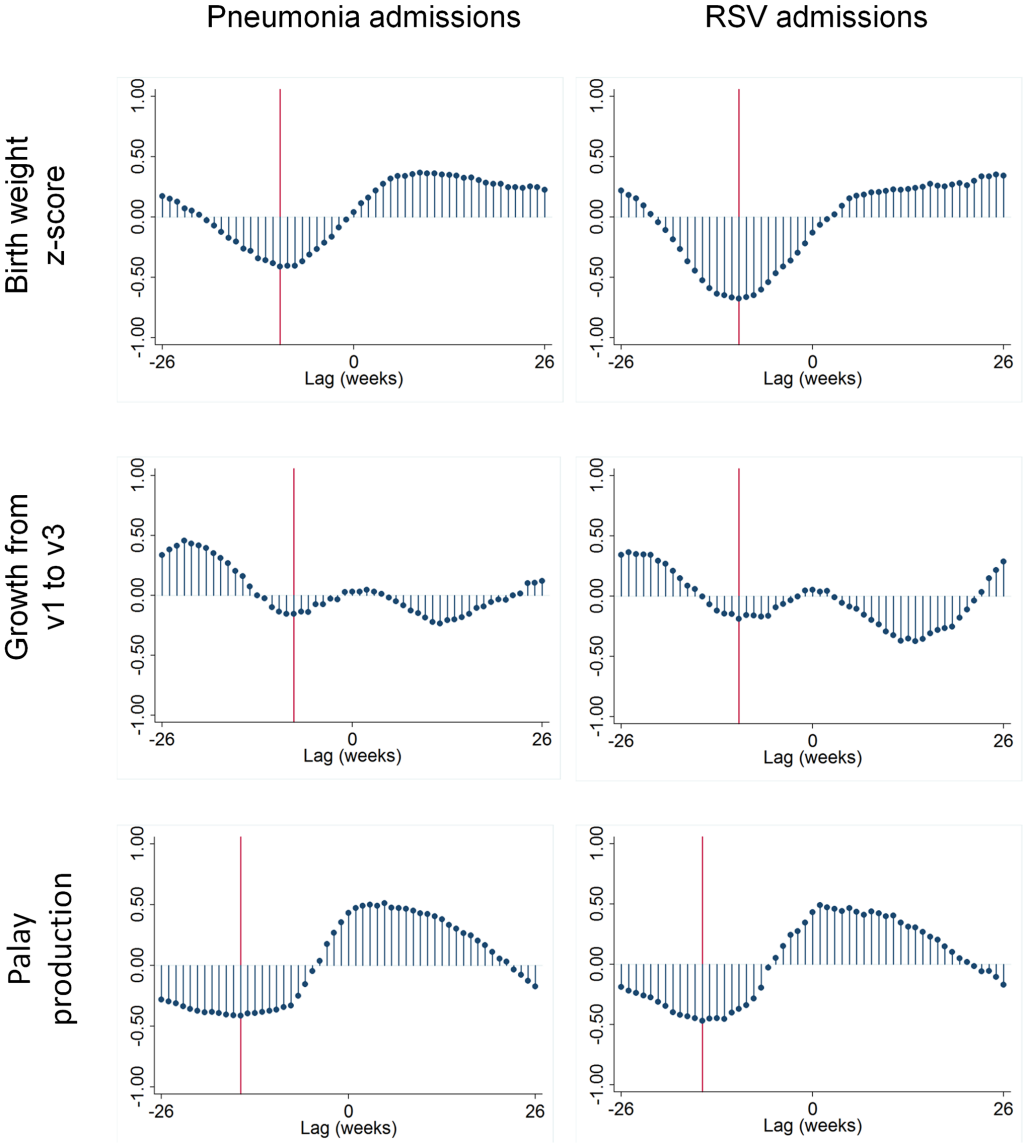
Weekly cross-correlations of seasonal patterns of estimated birth weight z-scores, growth between first and third vaccination, and palay production, with weekly pneumonia and RSV admissions. A negative lag indicates exposure occurring before the outcome. Vertical reference lines indicate points of maximum correlation. Bohol, Philippines 2000 to 2003.

Figure 4 shows the seasonal patterns of estimated birth weight z-scores, relative growth between first and third vaccination and palay production, together with weekly pneumonia and RSV incidence. The time series of palay production suggests nutrition is poorest in the third calendar quarter. Birth weight and infant growth are both consistently reduced during the third calendar quarter. In addition, infant growth shows a consistent trough during the first calendar quarter. The cross-correlation analyses are shown in Figure 5. Seasonal variations in estimated birth weight z-scores showed a negative correlation with pneumonia and RSV admissions 10 weeks later. Seasonal variations in growth between first and third vaccination showed a negative correlation with pneumonia and RSV admissions 8–10 weeks later, and a second negative correlation with pneumonia and RSV admissions 12 weeks earlier (the latter due to the trough in infant growth in the first calendar quarter). Seasonal variations in palay production showed a negative correlation with pneumonia and RSV admissions 15 weeks later. Table 4 shows the results of pairwise correlation of weekly pneumonia and RSV admissions with the seasonal indices of growth and nutrition at the time lags highlighted in Figure 5. All of these correlations are statistically significant at the p = 0.05 level.

**Table 4:** Pairwise correlation of weekly pneumonia and RSV admissions with seasonal indices of growth and nutrition, at time lags of maximal correlation as highlighted in Figure 5.

	Pneumonia admissions		RSV admissions	
	Lag (weeks)	Correlation (95% CI)	Lag (weeks)	Correlation (95% CI)
Estimated birth weight z-score	−10	−0.41 (−0.53 to −0.27)	−10	−0.63 (−0.72 to −0.51)
Growth between first and third vaccination	−8	−0.18 (−0.33 to −0.03)	−10	−0.21 (−0.37 to −0.04)
Palay production	−15	−0.52 (−0.63 to −0.40)	−15	−0.55 (−0.66 to −0.42)

A negative lag indicates exposure occurring before the outcome.

## Discussion

We have confirmed in our study setting the well known association between poor growth and increased pneumonia incidence in individual infants. In addition, we have shown that at a population level in our study setting, seasonal undernutrition precedes the seasonal peak of pneumonia and RSV incidence in infants. The seasonal level association between poorer nutritional status in the third calendar quarter and subsequent pneumonia and RSV admissions was maximal at a lag of approximately 10 weeks. This lag is plausible considering the non-linear dynamics of infectious disease transmission, where seasonal scale increases in transmission may take weeks to force seasonal increases in disease incidence [20]. Mathematical modeling of pneumonia seasonality in this setting would be useful to further assess the plausibility of this lag. In addition, there is direct evidence to suggest this lag is biologically plausible. During the Dutch famine in the winter of 1944 to 1945, birth weights fell soon after the start of the famine, lagging up to one month behind the reduction in rations [21]. Infants born immediately prior to the famine had substantially increased mortality during the late post-neonatal period (age 90 to 365 days) but not before [22]. These data suggest a lag between the abrupt reduction in infant growth and increased infant mortality of two or more months, consistent with the lag found in our time series analysis.

Our time series of growth between first and third vaccination showed two troughs each year. One trough, occurring in the third calendar quarter, coincided with the yearly troughs in palay production and estimated birth weight z-scores. The other trough occurred in the first calendar quarter. This additional trough in growth may be due to diarrheal disease rather than poor nutrition. A recent study from the Philippines found that rotavirus prevalence was highest in infants aged three to five months, and most cases occurred between December and February [23]. This coincides almost exactly with the first quarter trough in growth between first and third vaccination. Poor child growth and infectious diseases have a synergistic relationship, where poor growth increases the risk of infectious diseases and infectious diseases (particularly diarrheal diseases) reduce growth [9]. It is interesting that this reduction in infant growth in the first quarter is not associated with an obvious subsequent peak in pneumonia or RSV admissions (note however there is a small secondary peak in pneumonia admissions early in 2001, and a similar secondary peak occurred early in 2004 although we have no growth data to compare to this). The most likely reason for the lack of an obvious increase in admissions following the first quarter trough in growth is that this trough occurs only in a small subgroup of the population. The available data only indicate this additional trough occurs in young infants, and it is unclear which, if any, other subgroups are affected. Further analysis of population subgroups, including modeling how variations in susceptibility in different subgroups affects overall transmission and incidence, will be necessary to assess this hypothesis.

Although our birth weight measure was estimated from weight for age z-scores at approximately two months of age, it is reassuring that the seasonal pattern of our estimated birth weight z-score time series is similar to that of actual birth weights from the earlier Bohol study. The correlation between birth weight and infant weight that we assumed when estimating our birth weight measure has been noted elsewhere: a study in Bangladesh found that weight at 12 months of age was still largely a function of birth weight [24]. A second study from Bangladesh found that the seasonality of birth weight was correlated both with nutrition in all children aged less than five years, and with rice availability [25]. This evidence reinforces our opinion that the lower estimated birth weights, reduced growth between first and third vaccination, and low palay production during the third calendar quarter indicate seasonal undernutrition in our study setting at this time.

In our individual level analysis we found that infant growth between first and third vaccination was associated with an increased risk of pneumonia only in the infants who were smaller than average at first vaccination. There are two possible explanations for this. Firstly, it is possible that larger infants have more energy reserves, and so can better withstand acute nutritional shocks. Secondly, it may be that our growth measure has poor validity in larger infants (as larger infants tended to have a drop in weight for age z-scores between first and third vaccination, while smaller infants tended to undergo ‘catch up’ growth). Our results are consistent with two previous studies examining the association between poor growth and hospital admissions in children: both studies found this association was stronger in children who were smaller to begin with [8], [14].

Household crowding is an independent risk factor for respiratory infection [26]. We controlled for this by including the number of children in the household in our individual level analysis. The number of children in the household also serves as a measure of socioeconomic status in the individual level analysis, however some residual confounding from socioeconomic status may remain. In contrast the seasonal level time series analysis should not be confounded by individual level socioeconomic status (however in many settings household income is under greatest pressure during the rainy season, and it is possible that health determinants other than nutrition may be affected at this time).

Several other environmental factors may play a role in driving seasonal patterns of respiratory infection in the tropics. There is good evidence to show that a larger number of children within the household is associated with an increased risk of RSV infection [26]. We found this same association in our study setting (Tables 1 to 3). This association is likely due to a combination of an increased chance of introduction of pathogens into the household, as well as increased crowding within the household. While the actual number of children in a household is unlikely to vary seasonally, it is hypothesised that household crowding may be increased during the rainy season as people stay indoors more to avoid bad weather (although this hypothesis has not yet been tested in tropical settings) [27]. Vitamin D deficiency appears to be associated with an increased risk of respiratory infection, and has been proposed as a driver of seasonal outbreaks of respiratory infections in temperate areas [28], [29]. Less data is available from tropical settings however. In a study in India children with acute lower respiratory infection had lower vitamin D levels than controls, suggesting that vitamin D deficiency could be a driver of respiratory infection incidence in tropical settings [30]. Whether or not seasonal vitamin D deficiency occurs in children in tropical settings is not clear. In tropical settings, sunshine hours are determined more by cloud cover than solar elevation, hence sunshine hours are generally lowest during the rainy season. In our study setting of Bohol, we have previously found the seasonal patterns of sunshine hours and pneumonia incidence to be negatively associated [31]. Studies directly measuring seasonal variations in vitamin D levels in children in the tropics will be necessary to investigate this hypothesis further. Pathogen survival and virulence may also vary seasonally due to environmental factors such as humidity and sunshine [32]. The survival of enveloped viruses such as RSV on environmental surfaces is generally better at lower humidity [33] (RSV is predominantly transmitted by direct and indirect contact rather than by aerosol [34]). Thus while humidity may be a driver of RSV transmission in temperate winters, it is unlikely to be a driver in tropical wet seasons. Seasonal variations in sunshine however may influence environmental virus survival in the tropics [27]. More than one factor is likely to be acting to drive seasonality, and the dominant driver is likely to vary from setting to setting. In many tropical settings (including our study setting) rainfall is high, sunshine is low, and nutrition is poorest all at the same time of year, making it difficult to tease apart the effects of these exposures. Closer investigation of tropical settings where respiratory infections occur outside of the rainy season may help to differentiate these effects.

The results from our study suggest that in addition to being an individual level risk factor for pneumonia, poor nutrition may act as a population level driver of seasonal pneumonia epidemics in the tropics. Further investigation of the seasonal level association, in particular the estimation of the expected lag between seasonal undernutrition and increased pneumonia incidence, will be required to assess causality.
